# Analysis of factors influencing post-pandemic sleep quality among hospital clinical staff in Haikou, China

**DOI:** 10.3389/fpsyt.2026.1791327

**Published:** 2026-07-22

**Authors:** Yingxiu Wang, Sizhe Xu, Jingzhu Guo, Timothy Noel Stephens

**Affiliations:** International Medical Center, Haikou People's Hospital, The First Affiliated Hospital of Hainan University, Haikou, China

**Keywords:** China, healthcare personnel, influencing factors, post-pandemic, sleep quality

## Abstract

**Introduction:**

Factors contributing to poor sleep quality among healthcare professionals in the period immediately following the COVID pandemic are incompletely understood.

**Methods:**

Clinical staff from one tertiary hospital in Haikou, China, were invited in April 2023 via social media groups to complete a 33-item questionnaire comprised of demographic data, factors influencing sleep quality, and the Chinese versions of the Pittsburgh Sleep Quality Index (PSQI), the simplified Generalized Anxiety Disorder Scale (GAD-2), and the simplified Patient Health Questionnaire (PHQ-2). Data were analyzed using descriptive and multiple linear regression statistics.

**Results:**

The prevalence of poor sleep quality (PSQI score ≥7) was 55.8% (293/525). Sleep quality was significantly poorer in nurses as compared to physicians and other clinical staff (p=0.001). Staff with higher levels of education reported better sleep quality (p=0.039). Anxiety and depression symptoms were associated with poor sleep quality, prolonged sleep onset, decreased total sleep time, decreased sleep efficiency, sedative-hypnotic use, and daytime functional impairment (p<0.001). Multiple linear regression analysis found independent statistically significant associations between sleep quality and profession type, education level, night shift work, and anxiety and depression symptoms (p<0.05).

**Discussion:**

In the period immediately following the COVID pandemic, nurses were found to have poorer sleep quality than physicians and other hospital clinical staff. Night shift work, lower education level, and anxiety and depressive symptoms demonstrated the largest associations with poor sleep quality. Further research should investigate methods for identifying hospital clinical staff at risk of poor sleep and its consequences and effective preventive and mitigation measures.

## 1 Introduction

Sleep is an essential physiological activity for humans (1). In recent decades, lifestyle changes related to societal trends and economic development have been coupled with an increase in the prevalence of sleep disorders. Sleep disorders negatively impact physical, psychological, emotional, and social functioning, and are common among all age groups (2, 3).

Healthcare personnel, and in particular hospital clinical staff, are essential workers who may be at increased risk of sleep disorders due to busy clinical workloads, shift work, and high mental pressure. Poor sleep quality and inadequate sleep among healthcare professionals negatively impact clinical safety and quality (4, 5). Frequent overtime and night shifts resulting in insufficient sleep may lead to adverse effects on physical health and increased risk of accidents (6). Furthermore, frequent night shifts and lack of sleep among hospital clinical staff have been found to be risk factors associated with burnout (7).

The COVID pandemic placed unprecedented pressure on healthcare workers worldwide. In response to the public health threat posed by COVID, China imposed a broad array of strict prevention and control measures, including city-wide ‘lock-down’ restrictions on movement and rapid deployment of healthcare personnel to conduct mass nucleic acid testing and vaccination campaigns. Many healthcare workers spent long hours working in isolation wards and critical care units while facing the physical demands of wearing personal protective equipment and psychological challenges such as risk of infection, disruption of routine, and separation from loved ones and colleagues. The lifting of pandemic control measures in China in December 2022 was followed by a surge of COVID cases and hospitalizations through early 2023, necessitating hospital staff to work overtime while caring for many critically ill patients (8).

Multiple studies on factors influencing sleep quality among frontline medical workers in China were conducted during the pandemic. In 2020, Chen et al. (9) found a 47.8% prevalence of sleep disorders among frontline medical staff in one province. Cai et al. (10) found poor sleep quality among hospital personnel during the pandemic, with nurses, female staff, and those with intermediate professional titles being more likely to report insomnia. However, less is known about the sleep quality of healthcare workers during the period immediately following relaxation of COVID pandemic restrictions in China in early 2023. A 2023 survey of 622 clinical staff across China found that 68% had poor sleep quality (Pittsburgh Sleep Quality Index score ≥7) (11). However, few studies have clarified factors influencing sleep quality among healthcare personnel in the period immediately after the COVID pandemic.

This study aimed to evaluate the sleep quality of clinical staff at one hospital in Haikou, China, and factors influencing their sleep quality in the period immediately following normalization of COVID pandemic prevention and control measures. It is hoped that this study will serve as a reference for future research into means of identifying healthcare personnel at increased risk of poor sleep quality and its consequences when facing future pandemics or similar emergencies.

## 2 Materials and methods

A cross-sectional survey was conducted from April 1 to 30, 2023, among full-time clinical staff from a single public tertiary hospital in Haikou, China. The chosen hospital was designated as a COVID treatment hospital in September 2022 and treated a high proportion of local COVID patients in early 2023 after pandemic restrictions were lifted. Participants were invited via WeChat social media groups to complete a questionnaire using an online mini program (Wenjuanxing). All participants gave informed consent prior to voluntary participation.

### 2.1 Questionnaire design

A 33-item questionnaire designed by the investigators based on clinical experience informed by the literature included 8 demographic items, 3 items pertaining to factors influencing sleep quality (night shift work, tea or coffee consumption, and exercise habits), and items from three validated simplified Chinese questionnaires: the Pittsburgh Sleep Quality Index (PSQI), simplified Generalized Anxiety Disorder Scale (GAD-2), and simplified Patient Health Questionnaire (PHQ-2).

The Pittsburgh Sleep Quality Index (PSQI) was used to evaluate participants’ sleep quality via 19 self-rated items measuring 7 components: sleep quality (A), sleep latency (B), sleep duration (C), sleep efficiency (D), sleep disturbances (E), sedative-hypnotic use (F), and daytime functional impact (G) (12). Each component is given a score of 0-3, with a score ≥ 2 considered an abnormal result. The total PSQI score ranges from 0 to 21, with higher scores indicating poorer sleep quality. This study used a cut-off score of ≥ 7 for poor sleep quality (13).

The GAD-2, the simplified two-item version of the original Generalized Anxiety Disorder Scale (GAD-7), was used to assess anxiety symptoms: “Over the past two weeks, I have often been worried about events and activities around me” and “Over the past two weeks, I have been unable to stop worrying about life events and activities” (14). The total score ranges from 0 to 6, with a higher score indicating more severe anxiety symptoms. This study used a cut-off score of ≥ 3 for the presence of anxiety (15).

The PHQ-2, the simplified two-item version of the original Patient Health Questionnaire (PHQ-9) was used to assess depression symptoms: “Feeling low/loss of pleasure in the past two weeks” and “Lack of motivation/interest in doing things in the past two weeks”. A 2022 study evaluating the Chinese PHQ-2 scale found a sensitivity of 84% and specificity of 87% for depression (16). The total score ranges from 0 to 6, with higher scores indicating more severe depression. This study used a cut-off score of ≥ 3 for the presence of depressive symptoms.

### 2.2 Statistical analysis

Statistical analysis of data was performed using SPSS 25.0 software. Data followed a normal distribution, and student’s t-test was performed for comparisons between two groups. One-way ANOVA and LSD pairwise comparison were performed for comparisons between three groups, with results expressed as mean values ± standard deviation. Pearson correlation was used for correlation analysis. The total PSQI score was analyzed using multiple linear regression with “sleep quality” as the dependent variable and “profession”, “education level”, “night shift work”, “depression symptoms”, and “anxiety symptoms” as independent variables. Variables including “gender”, “marital status”, and “monthly income level” which did not demonstrate statistically significant correlations were not included in the multiple linear regression model. A p value <0.05 was considered a statistically significant difference.

## 3 Results

In total, 525 out of 806 distributed questionnaires (response rate 65.1%), 100% of which were complete, were considered valid for inclusion in analysis. Survey respondents included 94 males (17.9%) and 431 females (82.1%); average age of 42.31 years old (22–61 years); 432 respondents (82.29%) were married with children. There were 125 doctors (23.81%), 280 nurses (53.33%), and 120 other clinical staff (22.86%). The majority held intermediate level professional titles (50.67%), had a bachelor’s degree level of education (77.14%), and worked night shifts (61.59%). See **Table 1**.

**Table 1:** Basic characteristics.

Characteristic	N	Frequency (%)
Gender		
male	94	17.90
female	431	82.10
Marriage status		
yes	432	82.29
no	93	17.71
Profession type		
doctor	125	23.81
nurse	280	53.33
other (e.g. pharmacist)	120	22.86
Professional title		
junior	156	29.71
intermediate	266	50.67
senior	103	19.26
Education level		
associate’s degree	38	7.24
bachelor’s degree	405	77.14
master’s degree	68	12.59
doctoral degree	14	2.67
Night shift work		
Yes	323	61.59
No	202	38.48
Monthly income level (CNY)		
<5000	91	17.33
5000-9999	375	71.43
10000-14999	49	9.33
>15000	10	1.90
Prior medical care for sleep problem		
yes	47	8.95
no/never	478	91.05

### 3.1 Sleep quality

The prevalence of poor sleep quality (PSQI score ≥ 7) was 55.80% (293/525). The top three sleep quality component scores with abnormal scores (≥ 2) were sleep latency (59.43%), sleep duration (57.90%), and daytime dysfunction (57.52%). See **Table 2**.

**Table 2:** PSQI sleep quality factor component scores.

Component	Score (mean ± SD)	Score = 0 (N)	Score = 1 (N)	Score = 2 (N)	Score = 3 (N)	Abnormal score* (%)
A Sleep quality	1.53 ± 0.83	55	194	217	59	52.57%
B Sleep latency	1.71 ± 0.99	71	142	180	132	59.43%
C Sleep duration	1.56 ± 0.74	45	176	269	35	57.90%
D Sleep efficiency	0.41 ± 0.79	388	85	28	24	9.90%
E Sleep disturbance	1.34 ± 0.66	31	310	159	25	35.05%
F Sleep medication	0.19 ± 0.62	474	16	22	13	6.67%
G Daytime dysfunction	1.67 ± 0.94	62	161	191	111	57.52%

PSQI, Pittsburgh Sleep Quality Index; * abnormal score defined as ≥2.

### 3.2 Factors affecting sleep quality

#### 3.2.1 Night shift work

Clinical staff who worked night shifts were found to have significantly poorer sleep quality (PSQI score 8.7 ± 3.6) than those who did not (7.9 ± 3.6) (p = 0.012).

#### 3.2.2 Profession type

A statistically significant difference in sleep quality was found between nurses, doctors, and other professions (p = 0.001). Sleep quality was significantly poorer among nurses as compared to doctors (p<0.05) and other profession types (p<0.05). See **Table 3**.

**Table 3:** Factors influencing sleep quality.

Variable	Categories	PSQI score (mean ± SD)	t/F	p value
gender	male	7.94 ± 3.49	-1.385	0.167
	female	8.51 ± 3.66		
marital status	married	8.42 ± 3.66	0.18	0.857
	unmarried	8.34 ± 3.51		
night shift work	yes	8.72 ± 3.6	2.507	0.012*
	no	7.91 ± 3.63		
profession type	doctor	8.11 ± 3.06	7.173	0.001*
	nurse	8.93 ± 3.9		
	others	7.5 ± 3.32		
professional title	junior	8.45 ± 3.74	1.407	0.24
	intermediate	8.63 ± 3.72		
	vice-senior	7.75 ± 3.17		
	senior	7.81 ± 3.23		
education level	associate’s degree	9.47 ± 3.99	2.804	0.039*
	bachelor’s degree	8.48 ± 3.7		
	master’s degree	7.5 ± 2.71		
	doctoral degree	7.64 ± 3.67		
monthly income (CNY)	<5000	8.46 ± 4.08	0.735	0.531
	5000-9999	8.47 ± 3.54		
	10000-14999	8.1 ± 3.68		
	≥15000	6.9 ± 2.03		
tea/coffee intake (cups/day)	0	8.58 ± 3.6	1.290	0.277
	1-2	7.96 ± 3.74		
	3-4	8.96 ± 3.09		
	≥5	8.64 ± 3.73		
exercise (times/week)	0	8.48 ± 3.56	0.378	0.769
	1-2	8.29 ± 3.63		
	3-4	8.8 ± 4.02		
	≥5	7.95 ± 3.83		

*p<0.05; PSQI, Pittsburgh Sleep Quality Index.

#### 3.2.3 Education level

Staff with lower education levels, a bachelor’s degree or below, had poorer sleep quality (as indicated by total PSQI score) than those with a master’s degree or higher (p = 0.039).

#### 3.2.4 Anxiety and depression symptoms

The proportion of respondents with anxiety and depression symptoms was 48.81% (124/525) and 17.14% (90/525), respectively. Pearson correlation analysis demonstrated a statistically significant positive correlation between the presence of anxiety and/or depression symptoms and all seven PSQI sleep quality component scores (p<0.001). See **Table 4**.

**Table 4:** Pearson correlation analysis of PSQI sleep quality component scores and anxiety/depression symptoms.

Component	Anxiety symptoms		Depression symptoms	
	r	p value	r	p value
A Sleep quality	0.461	<0.001*	0.441	<0.001*
B Sleep latency	0.305	<0.001*	0.313	<0.001*
C Sleep duration	0.293	<0.001*	0.300	<0.001*
D Sleep efficiency	0.167	<0.001*	0.158	<0.001*
E Sleep disturbance	0.425	<0.001*	0.398	<0.001*
F Use of sleep medication	0.256	<0.001*	0.233	<0.001*
G Daytime dysfunction	0.542	<0.001*	0.553	<0.001*

*p<0.05.

### 3.3 Factors influencing sleep quality: multiple linear regression analysis

Using total PSQI score as the dependent variable, and “profession type”, “educational level”, “night shift work”, “anxiety symptoms”, and “depression symptoms” as independent variables, multiple linear regression analysis found “lower education level”, “night shift work”, “anxiety symptoms”, and “depression symptoms” were correlated with statistically significant differences in sleep quality, with (p <0.001, <0.002, <0.001, and <0.001). The F-value of the regression model was 61.425 (p<0.001), indicating significant overall fit of the multiple linear regression model. After model adjustment, R^2^ = 0.366 indicated a good explanatory power of the model. Results of multicollinearity diagnosis showed that the tolerance range of each variable was 0.511~0.976, and the variance inflation factor (VIF) of all independent variables was between 1.024 and 1.958. The degree of multicollinearity between variables was low, and the regression model parameter estimation results were stable and reliable. See **Table 5**.

**Table 5:** Multiple linear regression analysis of factors influencing sleep quality.

Variable	Unstandardized coefficient		Standardized coefficient	t	p value		
	B(95% CI)	SE	β			Tolerance	VIF
constant	8.701 (7.117- 10.285)	0.806		10.790	<0.001		
profession	-0.258 (-0.640- 0.124)	0.194	-0.049	-1.325	0.186	0.903	1.108
education level	-0.930 (-1.404- -0.456)	0.241	-0.140	-3.852	<0.001*	0.922	1.084
night shift work	0.612 (0.096- 1.127)	0.262	0.082	2.331	0.020*	0.976	1.024
anxiety	0.765 (0.546- 0.985)	0.112	0.334	6.864	<0.001*	0.512	1.952
depression	0.788 (0.537- 1.039)	0.128	0.300	6.170	<0.001*	0.511	1.958

*p<0.05; SE, standard error; CI, confidence interval; VIF, variance inflation factor.

## 4 Discussion

The special nature of work in healthcare places significant demands on clinical professionals. Healthcare professionals face heavy work pressures, intense and urgent clinical challenges, long work hours and shift work, the need to continuously update professional knowledge and skills, the stress of caring for suffering and dying patients, and difficult interactions with patients and their families, physical and psychological stressors that could contribute to poor sleep quality. A 2015 survey by Tian et al. (17) among 1200 hospital clinical staff in Heilongjiang, China, found an overall 45.77% prevalence of sleep disorders, with an even higher prevalence of 64.70% among nurses.

A repeated cross-sectional general population study in China during the COVID pandemic found an increased prevalence of anxiety, depression, and insomnia symptoms (17.3% -22.2%) during a period of resurgent cases in 2022 which persisted into mid-2023 after the lifting of pandemic control measures (14.5% -18.6%), demonstrating the ongoing impact of the pandemic on mental health in the general population (18). Sleep quality among healthcare professionals appears to have similarly worsened post-pandemic. Roncero et al. (19) studied the sleep quality of 1,121 medical staff in Spain after the pandemic, finding significant levels of moderate insomnia (23%), severe insomnia (3%), depression (28%), and anxiety (33%), with those working night shifts displaying significantly poorer sleep quality (mean Insomnia Severity Index score 11.3 ± 0.9 versus 8.8 ± 0.3, p<0.001).

The current study focused on the sleep quality of hospital clinical staff in Haikou, China, during the period immediately following lifting of COVID pandemic control measures in early 2023, finding high prevalences of poor sleep quality (55.8%) and anxiety (48.8%). These findings are noticeably higher than data from a June 2023 study in which 18.0%, 16.4%, and 20.5% of Chinese clinical staff reported anxiety, depression, and insomnia symptoms (20). This difference may be related to this study’s focus on a designated COVID treatment hospital, where the majority of hospital staff participated in front-line pandemic work. Furthermore, hospital staff faced high work pressure and taxing workloads just prior to this study during a peak in COVID hospitalizations in early 2023 that occurred shortly after lifting of pandemic measures in December 2022 (8). At the time of this study, many clinical personnel had just returned to their regular clinical duties immediately after completing frontline pandemic-related assignments. One could speculate that such a transition, which did not allow for interim rest, could potentially contribute to physical and mental exhaustion and consequently poor sleep quality.

In terms of profession type and work hours, the current study found that clinical staff working night shifts, and in particular nurses, had poorer sleep quality than other clinical staff, congruent with findings from multiple studies before and during the COVID pandemic. A 2014 survey of tertiary hospital nurses in Bangalore, India, found 53.8% had sleep problems (21). A 2020 survey of 1168 tertiary hospital nurses in Shiyan, China, found 25.7% had poor sleep quality (22). Su, Feng et al. (23) surveyed 506 nurses from one hospital in Guangdong, China, during the pandemic from 2022 to 2023. They found that 96.44% had reduced sleep quality, of which 42.09% had moderately reduced (PSQI score 11-15) and 21.34% had severely reduced sleep quality (PSQI score 16-21). A national study of 2065 nurses involved in pandemic frontline work found over half (52.7%) of nurses had sleep disorders (24). That study found nursing professional titles, personality traits, COVID-19 infection status, and exercise frequency were statistically significantly related to sleep disruption. The current study found no significant improvement in sleep quality among nurses during the period immediately after the pandemic. Furthermore, working night shifts demonstrated a significant negative impact on sleep quality, perhaps due to circadian rhythm disruption at a time when nurses needed to restore normal sleep patterns after a period of intense stress.

In terms of education level, the current study found that clinical staff with a master’s degree or higher reported significantly better sleep quality compared to those with a bachelor’s degree or below, congruent with results from multiple studies in China. Chen et al. (25) found that Beijing tertiary hospital medical staff with a master’s degree or above had a lower likelihood of sleep disorders. Wang et al. (26) found that among medical staff in Shanghai, a lower education level was associated with poorer sleep quality and insomnia (p=0.004), and female healthcare workers were more likely to experience anxiety symptoms than their male counterparts (OR = 2.772, p=0.008). These findings could relate to the beneficial effects of higher financial compensation on mitigating stress levels, as men and those with higher education levels are generally more well paid. However, in the current study, monthly income level was not significantly correlated with better sleep quality. In contrast, clinical staff with lower education levels may have lacked effective coping skills when faced with the complex professional, personal and family-related stressors during the COVID pandemic, resulting in higher levels of psychological distress and poor sleep quality.

Regarding the relationship between sleep quality and mental health, this study found that poor sleep quality among clinical staff immediately after the COVID pandemic was positively correlated with depression and anxiety symptoms. The high prevalence of mental health problems among Chinese healthcare professionals found in this and other studies underscores the need to further address this important contributing factor to poor sleep quality. A 2021 survey by Zhang et al. (27) among 659 female medical staff in Beijing found a high proportion reported obvious symptoms of stress (60.1%), depression (45.1%), and anxiety (19.1%). A 2023 cross-sectional study of 645 medical staff from four hospitals in Sanya, China, found a high prevalence of depression (42.7%), with similarly high prevalences of depression combined with anxiety (23%) and sleep disorders (34.5%) (28). The prevalence of depression among female staff, nurses, unmarried or single individuals, and shift workers was significantly higher than among male staff (48.3% vs 27.1%, OR = 2.512) and doctors (55.2% vs 26.7%, OR = 3.388) in that study. Multiple logistic regression analysis found that nurses were at higher risk for depression, as were staff with anxiety and sleep disorders. The results of the current study also showed a significant correlation between sleep quality and anxiety and depression. However, in this study, lower education level and night shift work were found to have independent associations with poor sleep quality on multiple linear regression analysis, whereas gender or profession type did not. This result concurs with findings from a study in Huizhou, China, which did not find higher risks of anxiety, depression or insomnia among female healthcare staff (29). These findings underscore that anxiety and depression are important issues to be identified and addressed among all healthcare staff of both genders, regardless of education level or profession type.

### 4.1 Limitations

The cross-sectional design of this study did not allow for the evaluation of pre- and post-pandemic changes over time in sleep quality, anxiety, and depression symptoms, limiting the ability to discriminate between effects of pandemic stress or exhaustion and pre-existing influencing factors. Future research should investigate trends in sleep quality, anxiety, and depression symptoms among healthcare professionals in the years after the pandemic to determine if a tail effect is present. Furthermore, the validated scales used in this study depend upon subjective reporting by participants, which may skew results due to recall bias. Future research should consider adding objective measures of sleep quality such as actigraphy data to clarify the relationship between subjective assessment and sleep efficiency. Additionally, this study only obtained data from one hospital limiting generalizability of findings. Data from larger multi-center samples across multiple hospitals would enhance generalizability of results. The small sample size of non-physician non-nurse clinical personnel also did not allow for subgroup analysis of other clinical personnel. Finally, whereas this study investigated the correlation between sleep quality and anxiety, depression, and influencing factors such as night shift work, future studies should investigate the effects of other factors related to sleep quality including burnout, personal history of COVID infection, and grief related to pandemic-related deaths of friends or relatives.

### 4.2 Implications for practice

This study provides further evidence of the lingering effects of the COVID pandemic on the sleep quality and mental health of hospital clinical staff in the period immediately following the pandemic, underscoring the need for further research into effective means of identifying and supporting staff at risk of decreased daytime functioning and burnout. Research into post-traumatic effects of emergencies and pandemics on the mental health and sleep quality of healthcare workers has found a significantly increased risk of burnout and negative impact on functioning, which could impact patient safety (30). A 2021 systematic review by d’Ettore et al. (31) found that younger age, limited work experience, female gender, heavy workload, and lack of training and social support were found to be predictors of post-traumatic stress symptoms in healthcare workers dealing with the COVID pandemic. Personal and systemic factors, notably a tendency to not seek out emotional support and stigma regarding seeking psychological support, also increased the risk of pandemic-related psychological distress and insomnia among healthcare workers (32). A meta-analysis of pandemic-related post-traumatic stress disorder among health professionals from both the SARS 2003 and COVID 2019 pandemics pointed out the importance of gaining a comprehensive understanding of both immediate- and delayed-onset impacts of pandemic-related stressors on the mental wellbeing of clinical staff (33). A recent post-pandemic study among Iranian healthcare workers found that post-traumatic feelings of helplessness and insufficient support systems were related to intention to leave the healthcare profession altogether (34). Furthermore, mechanisms by which sustained neuroinflammatory effects of COVID infection on mood and cognitive function interact with unique challenges faced by healthcare workers including exposure to patient deaths, moral injury, and work-related exhaustion require additional investigation (35, 36).

Given the potential negative impact of poor sleep among healthcare professionals on clinical quality and patient safety, further research is also needed into effective risk mitigation strategies that healthcare institutions can adopt. For example, development of comprehensive Fatigue Risk Management Systems that ensure adequate sleep opportunities for employees, confirm adequate sleep has been obtained and that employees are fit for duty, and checklists to detect behavioral symptoms of fatigue have been used broadly in other industries such as aviation and transport but have not yet been widely adopted in healthcare settings (37).

Evolving evidence regarding interventions to ameliorate the effects of night shift work on healthcare professional sleep quality has highlighted the need for both individual-level and institutional-level strategies. For example, hospital management could ensure staff have adequate opportunities for sleep on days off by allowing flexible work schedules and not requiring staff to work overtime or attend meetings or educational activities during time off (38). Policies that allow night shift nurses to take brief naps in a quiet environment within the workplace during a break could enhance cognitive function and work performance (39). For individuals suffering from poor sleep quality due to night shift work, proven interventions such as bright light therapy given 1 to 2 hours before awakening could enhance sleep quality and reduce sleepiness and fatigue (40).

Finally, resilience training and education in stress coping skills could be developed by health systems to provide targeted psychological interventions for clinical staff identified as showing signs of poor sleep quality and associated mental health symptoms. A 2020 Cochrane systematic review found evidence that healthcare professionals who received resilience training based on mindfulness and cognitive-behavioral therapy showed lower levels of depression, stress or stress perception (41). Based on this study’s findings, those at higher risk of poor sleep such as those with lower educational levels could be potential targets for such programs.

## 5 Conclusion

This cross-sectional single-center study of clinical hospital staff in Haikou, China, found high rates of poor sleep quality, anxiety, and depression symptoms in the period immediately following the COVID pandemic. In particular, nurses, those working night shifts, and staff with lower education levels were at higher risk for poor sleep quality. Additional studies are needed to assess the long-term impact on sleep quality and mental health of stressors faced by healthcare professionals during the pandemic and effective means of identifying clinical staff at risk of poor sleep quality during future health emergencies, with the goal of ensuring they can continue providing safe and high-quality care.

## Data Availability

The raw data supporting the conclusions of this article will be made available by the authors, without undue reservation.
